# A Case Report: Tragic Death in a Young Patient with Human Immunodeficiency Virus Due to Cryptococcal Meningitis

**DOI:** 10.7759/cureus.4652

**Published:** 2019-05-13

**Authors:** Ayomide Loye, Onyinye Gabriel, Xiao Chi Zhang

**Affiliations:** 1 Emergency Medicine, Thomas Jefferson University, Philadelphia, USA; 2 Pharmacy, Novartis Healthcare Private Limited, New Jersey, USA

**Keywords:** cryptococcus neoformans, intracranial pressure, lumbar puncture, emergency department

## Abstract

Cryptococcal meningitis is a systemic infection that can be seen in immunosuppressed patients. Altered mental status, somnolence, and obtundation are warning signs of poor prognosis or advanced disease processes.

We present a 23-year-old female with a past medical history significant for human immunodeficiency virus (HIV) obtained via vertical transmission who presented to the emergency department (ED) with a gradual onset of worsening headache over 10 days, with blurry vision, photophobia, nausea and vomiting, and progressive memory lapses. Her blood tests, chest plain radiograph, and non-contrast brain computed tomography (CT) were normal. In the ED, she developed a fever of 102°F and became more confused and agitated, with interspersed screaming and yelling. A lumbar puncture (LP) showed elevated white blood cell count and was positive for Cryptococcus neoformans; an opening pressure was unable to be obtained due to patient agitation. Despite prompt intravenous antibiotics and antifungal medications, her short, but tenuous hospital course involved declining mental status, requiring intubation and multiple therapeutic lumbar punctures, with an elevated opening pressure of up to 55 cm H2O. The patient suffered global ischemic encephalopathy and died on hospital day two.

This case highlights the rapid decompensation of a young immunocompromised patient with cryptococcal meningitis, as well as the importance of early disease management and consultation to neurology and neurosurgery services. An important paradigm difference for emergency medicine (EM) physicians in the management of increased intracranial pressure (ICP) in patients with cryptococcal meningitis is avoiding acetazolamide, mannitol, and steroids and considering the indication for neurosurgical interventions for severe cryptococcal meningitis.

## Introduction

Cryptococcal meningitis is a systemic mycotic infection caused by the yeast Cryptococcus [1] The pathogen Cryptococcus neoformans (C. neoformans) is predominately seen in immunosuppressed patients with human immunodeficiency virus (HIV) or acquired immunodeficiency syndrome (AIDS) [2]. C. neoformans infections can be subacute, chronic, and fatal and predominately affect the meninges and lungs [3-4]. Common symptoms include headache, nausea, vomiting, and malaise [5]. In patients that have advanced disease, altered mental status, somnolence, and obtundation are warning signs of poor prognosis [6].

## Case presentation

A 23-year-old female with a past medical history significant for human immunodeficiency virus (HIV) obtained via vertical transmission presented to the emergency department (ED) with a gradual onset of worsening headache over 10 days, with blurry vision, photophobia, nausea and vomiting, and progressive memory lapses. The patient was diagnosed at age two, but she had not taken any antiretroviral medications for the past year and she did not know her last CD4 count. The patient had visited two EDs previously, with unclear timelines, and was diagnosed with sinusitis and discharged home with antibiotics. Review of systems was negative for fever, nuchal rigidity, and weight loss.

On arrival to the ED, she had an initial temperature of 98.9°F, with a blood pressure of 94/79 mm Hg and a pulse rate of 79 beats/min. Her respiratory rate was 18 breaths/min with oxygen saturation at 100%. She did not have any focal neurological deficits and she was alert and oriented x 4. Her pupillary exam was significant for photophobia with equal and reactive 3 mm pupils bilaterally. Her blood tests, including complete blood count (CBC), comprehensive metabolic panel (CMP), urinalysis, and drugs of abuse screen were within normal limits. Electrocardiogram (EKG) showed normal sinus rhythm and chest plain radiograph was normal. A non-contrast brain computed tomography (CT) revealed no acute hemorrhage or lesion. Upon reassessment in the ED, she developed a fever of 102°F and became more confused and agitated, with interspersed screaming and yelling. A lumbar puncture (LP) was performed in the lateral decubitus position to analyze the cerebral spinal fluid (CSF), with opening pressure measurement due to concern for encephalitis; unfortunately, the patient was too agitated to safely obtain a reliable opening pressure. Results from the LP showed a cloudy appearing CSF, white blood cell (WBC): 22 (normal <5 per mm^3^), tube 1 red blood cell (RBC): 59 (normal <5 per mm^3^), % polymorphonuclear neutrophil (PMNs): 15% (normal 0% - 15%), % lymphocytes: 66% (normal >50%), glucose: 43 (normal >40 mg/dL), protein: 66 (normal <50 mg/dL). Gram stain was positive for Cryptococcus neoformans/budding yeast with a cryptococcal reactive antigen of 1:320 (normal <1:1). The patient was admitted to the medicine floor with a diagnosis of cryptococcal meningitis and was started on intravenous amphotericin B 250 mg every 24 hours and flucytosine 250 mg by mouth every six hours while in the ED, with recommendations from neurology and infectious disease consultation.

On hospital day one, the patient had a waxing and waning mental status. A repeat LP was performed by the inpatient team, which revealed an opening pressure of 55 cm H2O (normal <21cm). The decision was made to remove about 22 mL of fluid, with a closing pressure of 12 cm H2O, with documented mental status improvements. The CD4+ result was noted to be 15 cells/uL, confirming the diagnosis for acquired immunodeficiency syndrome (AIDS) [7]. Later that evening, while being transported for a repeat CT scan, the patient became acutely altered, lethargic, and bradycardic to the 40s with a blood pressure of 128/83 mmHg, necessitating a rapid response team (RRT). She was given one amp of D50 with a post-interventional point-of-care glucose level of 206 mg/dL and subsequent improvement of her mental status. Her repeat non-contrast brain CT showed no herniation and the patient was transferred to the intensive care unit (ICU).

**Figure 1:**
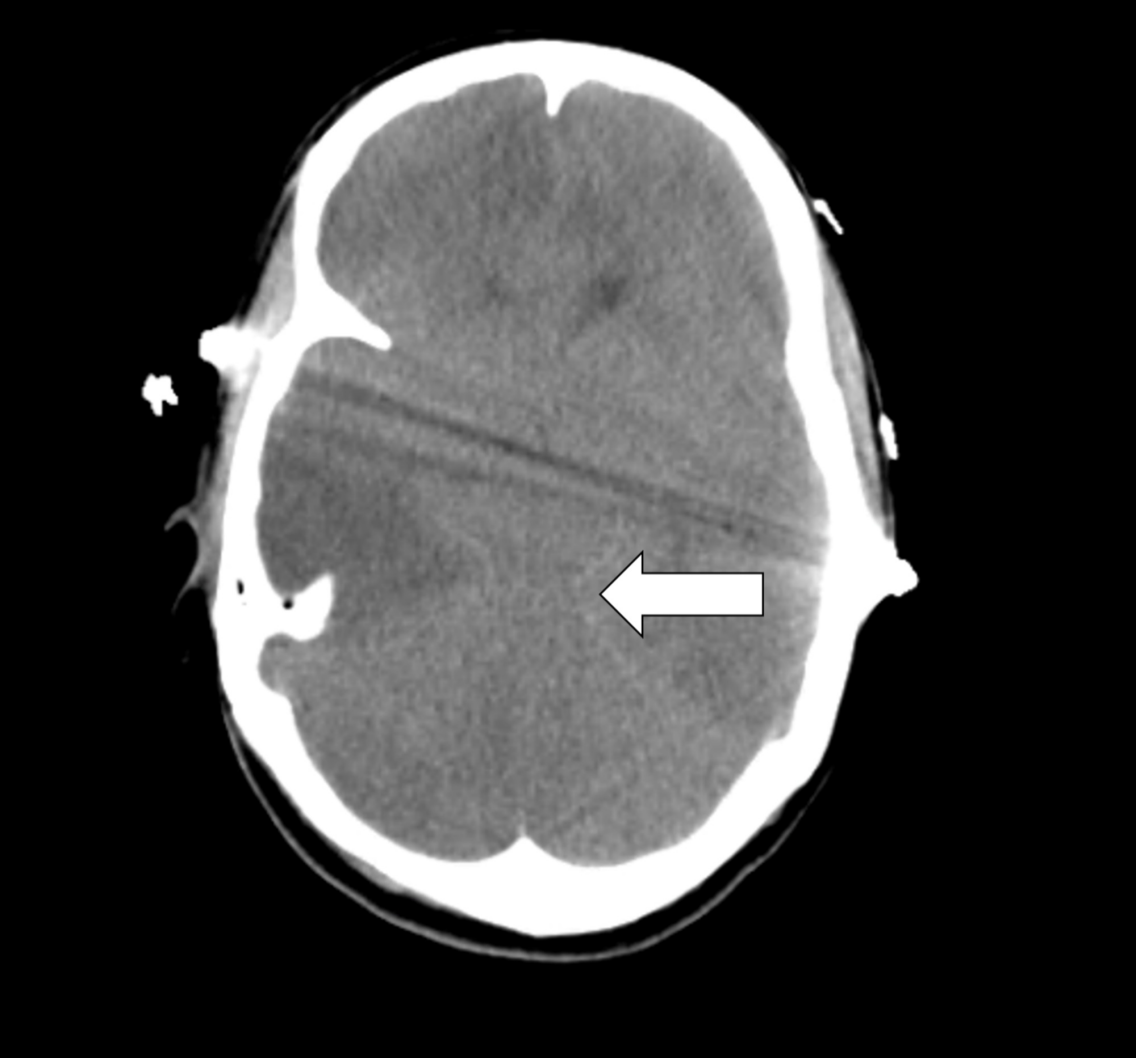
Post-intubation non-contrast computed tomography of the brain demonstrating loss of gray-white matter differentiation (arrow) with maintenance of perfusion of the thalami and deep gray matter concerning for global ischemic encephalopathy

On hospital day two, the patient became severely agitated, with worsening mental status changes, requiring 5 mg of intravenous haloperidol, followed by obtundation with decorticate posturing and sluggish pupils. The patient was immediately intubated for airway protection and a repeat non-contrast brain CT showed diffuse loss of gray-white matter differentiation with the maintenance of perfusion of the thalami and deep gray matter concerning for global ischemic encephalopathy (Figure 1). Post-intubation LP revealed an opening pressure of 55 cm H_2_O and 30 cc of bloody fluid were removed to reduce intracranial pressure. Shortly after the procedure, the patient became tachycardic and a closing pressure was unattainable. She went into pulseless electrical activity (PEA) cardiac arrest with a return to spontaneous circulation after 30 seconds of chest compressions. Her post-arrest exam was significant for absent brainstem reflexes and the patient was ultimately declared brain dead in the following week.

## Discussion

This is a tragic case report of cryptococcal neoformans meningitis in a young female patient with AIDS, with rapid decompensation due to disease progression and increased intracranial pressure.

Cryptococcal meningitis is a well-known but highly dangerous opportunistic HIV-related infection of the spinal meninges and brain, with high mortality and morbidity. Globally, the incidence of cryptococcal meningitis in HIV-infected patients ranges from 0.04% to 12% per year, with approximately 624,700 deaths a year within three months of diagnosis [1]. Approximately half of the surviving patients developed long-term neurological deficits [2]. There was a correlation with a delay in the time of onset from symptoms to diagnosis, a delay in time from presentation to diagnosis, female gender, and raised increased intracranial pressure (ICP) with having poor neurological outcomes. Immunocompromised patients with Cryptococcus gatti meningitis with CSF antigen titer > 256 experienced higher mortality rates and worsening neurological sequela [2,8]. Our patient had a cryptococcal titer of 1:320.

The treatment of cryptococcal meningitis is divided into the induction phase, consolidation phase, and maintenance phase. The induction phase consists of combination therapy with intravenous amphotericin B (0.7-1.0mg/kg per day) plus oral flucytosine (100 mg/kg per day divided into four doses) for at least two weeks for rapid sterilization of cryptococcal from the CSF [9-10]. The role of flucytosine has been suggested to be the prevention of relapse when used during the induction phase [6]. After a minimum of two weeks of induction therapy with negative CSF cultures and clinical improvement, the consolidation phase begins with fluconazole (400 mg (6 mg/kg) per day orally) for a minimum of eight weeks [8-9]. Antiretroviral therapy (ART) should be started on patients with HIV during the consolidation phase for immune restoration [10]. The maintenance phase consists of fluconazole (200-400 mg/kg per day) for about six to 12 months for relapse prevention. Maintenance therapy can be discontinued once CD4>100 cell/uL [11].

A major complication of cryptococcal neoformans meningitis is elevated intracranial pressure. This has been suggested as the main cause of early mortality even after the onset of induction therapy, as evident by our patient. The pathophysiology of elevated ICP in Cryptococcus neoformans has been hypothesized as the decreased absorption at the arachnoid villi secondary to increased CSF viscosity or microscopic plugs from cryptococcal polysaccharide [12]. There are numerous treatment strategies for the management of increased ICP that range from serial lumbar punctures, external lumbar and ventricular drain placements, and ventricular or lumbar shunting [13]. Medical management of increased ICP with acetazolamide, mannitol, and steroids in HIV-associated cryptococcal meningitis is not recommended [9,11,14]. Dexamethasone has been associated with decreased fungal clearance and increased adverse event and disability in this population [15].

CSF drainage is a therapeutic method of ICP reduction, with a treatment endpoint of reducing the opening pressure to less than 20 cm H20, or in cases of very high ICP, a 50% reduction of the opening pressure [12]. Serial lumbar punctures are often the first procedure performed until the patient CSF pressures are stabilized [16-17]. Neurosurgery consultation for lumbar shunt should be considered if patients do not have relief of headache after lumbar puncture drainage, have persistently elevated pressure after at least three lumbar puncture taps, even with the initiation of antifungal therapy, or can no longer tolerate further lumbar punctures [18].

## Conclusions

This case highlights the rapid decompensation of a young immunocompromised patient with cryptococcal meningitis, as well as the importance of early disease management and consultation to neurology and neurosurgery services. Patients at risk for developing cryptococcal meningitis should be treated immediately with antibacterial and antifungal agents in addition to obtaining and observing the opening CSF pressure throughout the patient’s hospitalization, as increased CSF pressure is the main cause of early mortality. An important paradigm difference for emergency medicine (EM) physicians in the management of increased ICP in patients with cryptococcal meningitis is avoiding acetazolamide, mannitol, steroids, and serial lumbar punctures, and considering the indication for neurosurgical interventions for severe cryptococcal meningitis.
